# Origin of the near-room temperature resistance transition in lutetium with H_2_/N_2_ gas mixture under high pressure

**DOI:** 10.1093/nsr/nwad337

**Published:** 2023-12-30

**Authors:** Di Peng, Qiaoshi Zeng, Fujun Lan, Zhenfang Xing, Zhidan Zeng, Xiaoxing Ke, Yang Ding, Ho-kwang Mao

**Affiliations:** Key Laboratory of Materials Physics, Institute of Solid State Physics, Hefei Institutes of Physical Science (HFIPS), Chinese Academy of Sciences, Hefei 230031, China; Science Island Branch, Graduate School of University of Science and Technology of China, Hefei 230026, China; Center for High Pressure Science and Technology Advanced Research, Shanghai 201203, China; Center for High Pressure Science and Technology Advanced Research, Shanghai 201203, China; Shanghai Key Laboratory of Material Frontiers Research in Extreme Environments (MFree), Shanghai Advanced Research in Physical Sciences (SHARPS), Shanghai 201203, China; Center for High Pressure Science and Technology Advanced Research, Shanghai 201203, China; Center for High Pressure Science and Technology Advanced Research, Shanghai 201203, China; State Key Laboratory of Superhard Materials, Institute of Physics, Jilin University, Changchun 130012, China; Center for High Pressure Science and Technology Advanced Research, Shanghai 201203, China; College of Materials Science & Engineering, Beijing University of Technology, Beijing 100124, China; Center for High Pressure Science and Technology Advanced Research, Shanghai 201203, China; Center for High Pressure Science and Technology Advanced Research, Shanghai 201203, China; Shanghai Key Laboratory of Material Frontiers Research in Extreme Environments (MFree), Shanghai Advanced Research in Physical Sciences (SHARPS), Shanghai 201203, China

**Keywords:** superconductivity, high pressure, lutetium hydride, resistance transition, metal-to-semiconductor/insulator transition

## Abstract

The recent report of room-temperature superconductivity at near-ambient pressure in nitrogen-doped lutetium hydride (Lu-H-N) by Dasenbrock-Gammon *et al.* [*Nature* 615, 244–250 (2023)] has attracted tremendous attention due to its anticipated great impact on technology. However, the results could not be independently reproduced by other groups worldwide in follow-up studies, which elicited intense controversy. Here, we develop a reliable experimental protocol to minimize the extensively concerned extrinsic influences on the sample by starting the reaction from pure lutetium loaded with an H_2_/N_2_ gas mixture in a diamond anvil cell under different pressures and temperatures and simultaneously monitoring the entire chemical reaction process using *in situ* four-probe resistance measurements. Therefore, we could repeatedly reproduce the near-room temperature upsurge of electrical resistance at a relatively early stage of the chemical reaction. However, the mechanism is suggested to be a metal-to-semiconductor/insulator transition associated with the structural modulation in the non-stoichiometric Lu-H-N, rather than superconductivity.

## Teaser

The near-room temperature resistance transition in the Lu-H-N compound is repeatedly reproduced, which is clarified to originate from a metal-to-semiconductor/insulator transition rather than superconductivity.

## INTRODUCTION

The transition of materials from a normal state to a superconducting state will be accompanied by a sudden change from finite electrical resistance to zero resistance, the hallmark and one of the most desirable properties of a superconductor. The first discovery of superconductivity was made in mercury with zero resistance back in 1911 when it was cooled down to an extremely low temperature of ∼4 K. Since then, the quest for superconductors existing at higher temperatures has attracted enduring efforts [1]. Applying high pressure has been proposed and evidenced to be a promising and effective way for many materials to elevate their superconductive transition temperatures (*T*_c_) even close to room temperature, which, however, typically requires extreme pressures up to tens or hundreds of GPa, far from practical application conditions [2–15]. The recent report of superconductivity on nitrogen-doped lutetium hydride (Lu-H-N) with a maximum *T*_c_ of 294 K at only 1 GPa represents a significant step forward in approaching a realistic superconductivity era [16]. However, the non-reproducibility of the work of other researchers who followed the method of synthesis for Lu-H-N in Ref. [16] and the inscrutable low success rate (35%) in synthesizing the right sample even for the authors of Ref. [16] cast intense controversy and led to doubts on the claim from the entire scientific community [17–27].

To address the concerns accounting for the non-reproducibility, i.e. the claimed difficulties in controlling the reaction between lutetium and H_2_/N_2_ gas mixture to ensure the correct superconducting phase [14,16], in this work, we employ *in situ* electrical resistance measurements under high pressure for real-time monitoring of the entire reaction process between a piece of pure lutetium foil and H_2_/N_2_ gas mixture under various temperatures and pressures in a diamond anvil cell (DAC) [28]. On the one hand, we have a ‘clean’ chemical environment and can avoid potential sample contamination, oxidation, damage, or degradation of the synthesized Lu-H-N sample during transferring, manipulation, and loading, usually required for post-fabrication electrical resistance measurements under high pressure [29]. On the other hand, real-time resistance monitoring can help avoid missing any intermedium states/phases associated with the resistance jump during the reaction.

With this well-controlled experimental protocol, we reveal that the reaction between the pure lutetium foil and H_2_/N_2_ gas mixture eventually leads to the formation of an insulator with a resistance increase by up to 8 orders of magnitude compared with the initial pure Lu. The reaction rate strongly depends on the pressure and temperature conditions, which makes the synthesis of Lu-H-N difficult with arbitrary time, pressure, and temperature conditions and may account for the non-reproducibility of sharp resistance change in the Lu-H-N samples [17–26]. It is clarified that an abrupt resistance change can be repeatedly observed near room temperature in a dark blue sample only within the right time window at the very early stages of the reaction. *In situ* Raman spectroscopy and *ex situ* electron transmission microscopy (TEM) measurements both confirm the occurrence of the reaction between lutetium and H_2_/N_2_ gas mixture with new phases. However, the lack of both zero-resistance and magnetic-field suppression behavior of the resistance transition explicitly rules out the possibility of superconductivity. Instead, a reversible metal-to-semiconductor/insulator transition is suggested to result in the drastic resistance jump near room temperature.

## RESULTS AND DISCUSSION

**Figure 1.**
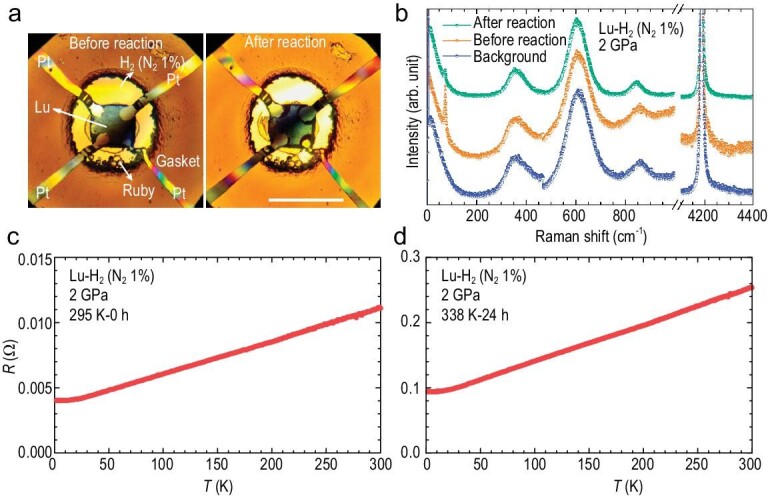
*In situ* characterization for the lutetium foil sample loaded with H_2_ (N_2_ 1%) gas mixture in a DAC at ∼2 GPa. (a) The optical microphotographs of the Lu foil sample just loaded (left, 295 K) and after a 24-hours reaction (right, 338 K) with H_2_ (N_2_ 1%) gas mixture in a DAC at 2 GPa with four platinum electrodes for *in situ* resistance measurement. The sample color, size, and chamber size all do not show obvious changes. The scale bar represents 100 μm. (b) Comparison of *in situ* Raman spectra of lutetium foil sample before and after reaction and the background signal of the H_2_ (N_2_ 1%) gas mixture at ∼2 GPa and 295 K. The background signal is mainly from H_2_ (∼360 cm^−1^, 610 cm^−1^, 850 cm^−1^). The signal from N_2_ is invisible due to the overlap of strong signals from the diamond anvil. One lattice vibration peak from the lutetium metal is visible at ∼74 cm^−1^. After reaction at 338 K and ∼2 GPa for 24 hours, the Lu signal remains, but with decreased intensity, and no visible new peaks emerge. Temperature dependence of resistance for lutetium foil sample immersed in the H_2_ (N_2_ 1%) gas mixture at ∼2 GPa during warming from 2 K to 300 K before (c) and after (d) the reaction at 338 K (65°C) for 24 hours.

First, we tried to reproduce the sudden resistance change of lutetium foil after its reaction with the H_2_/N_2_ gas mixture (the volume ratio is 99:1, hereafter denoted as H_2_ (N_2_ 1%)) at ∼2 GPa and 338 K (65°C) by following the experimental condition reported in Ref. [16]. With a four-probe circuit prepared as the lutetium foil sample loaded with H_2_ (N_2_ 1%) gas mixture in the DAC (Fig. 1a), the resistance and its temperature dependence can be obtained at any stage during the reaction by the Van der Pauw four-probe method [30] using the Physical Property Measurement System (PPMS, Quantum Design) at a good hydrostatic pressure condition [31]. Specifically, immediately after gas loading at ∼2 GPa and 295 K, the electrical resistance exhibits a typical metallic behavior with positive temperature dependence and obvious residual resistance below 15 K, as shown in Fig. 1c. After heating to 338 K (65°C) and holding there for 24 hours (Fig. 1d), the sample still remains in a regular metallic state but with much higher resistance compared with that of the initial as-loaded pure lutetium (Fig. 1c). The considerable increase in resistance is typical for hydrogenation of metals [32], which indicates that a chemical reaction indeed occurs between the lutetium foil and H_2_ (N_2_ 1%) gas mixture at the pressure-temperature conditions reported in Ref. [16]. However, no evidence of a superconducting transition near room temperature exists after the reaction. Moreover, there are no noticeable changes in the sample color, the sample chamber size also remains almost constant (Fig. 1a), and no new Raman peaks emerge (Fig. 1b), which, taken together, consistently confirms the reaction is sluggish and still extremely subtle at ∼2 GPa and 338 K even after holding for 24 hours. Therefore, tuning pressure and/or temperature is needed to promote the reaction with faster kinetics [33].

**Figure 2.**
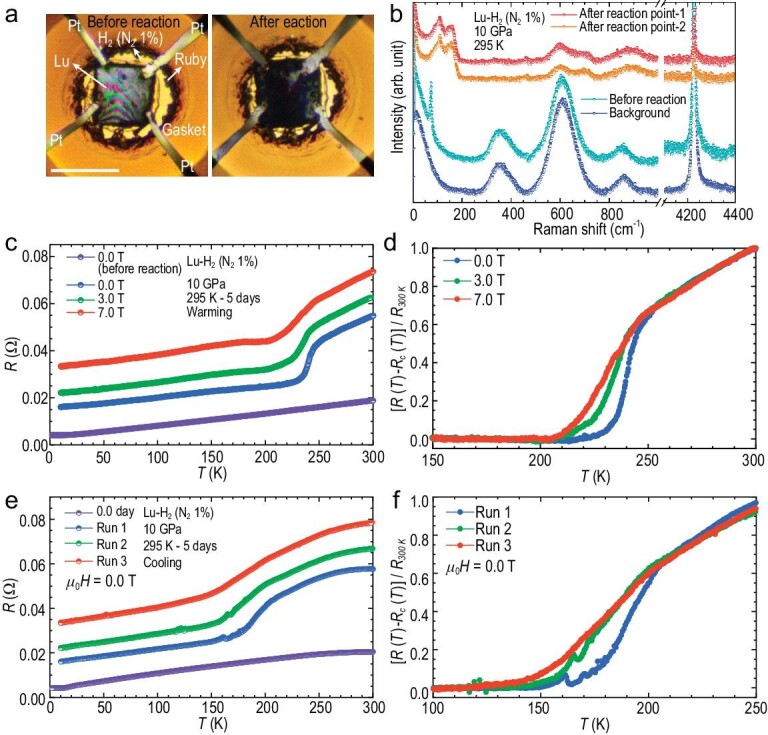
*In situ* characterization of the lutetium foil sample loaded with H_2_ (N_2_ 1%) gas mixture in a DAC at ∼10 GPa. (a) The optical microphotographs of the lutetium foil sample just loaded (left) and after 5 days of reaction (right) with H_2_ (N_2_ 1%) gas mixture at ∼10 GPa and 295 K with four platinum electrodes for *in situ* resistance measurement. The sample color and size changes and the sample chamber shrinkage (consumption of H_2_ (N_2_ 1%) gas mixture) indicate that a reaction occurs after holding at ∼10 GPa and 295 K for 5 days. The scale bar represents 100 μm. (b) Raman spectra of the lutetium foil sample before and after reaction and the background signal of the H_2_ (N_2_ 1%) gas mixture at ∼10 GPa and 295 K. The peak from the lutetium metal (∼74 cm^−1^) disappears, and a few new peaks emerge (e.g. between 150 cm^−1^ and 200 cm^−1^) after the reaction. Temperature dependence of raw resistance values during warming with different magnetic fields (c) and during cooling without magnetic fields (e) before (purple circles) and after 5-days reaction at ∼10 GPa and 295 K both confirm the emergence of the sudden resistance change associated with the reaction. Seemingly zero-resistance and magnetic suppression effects are observed in (d) and (f) after a linear background, *R*_c_(*T*), subtraction and normalization to the resistance at 300 K of the data in Fig. 2c and e, respectively. The sample images in (a) and part of the data (two curves at 0.0 T before and after the reaction) in (c) were from Ref. 28. (See online supplementary material for a colur version of this figure.)

Next, we reloaded the lutetium foil with the H_2_ (N_2_ 1%) gas mixture and a Van der Pauw four-probe circuit to explore higher pressure but lower temperature (∼10 GPa and 295 K) (Fig. 2a). The initial resistance-temperature curve (purple dots in Fig. 2c) still looks similar to that at ∼2 GPa and 295 K (Fig. 1c). (See online supplementary material for a colour version of this figure.) However, after 5 days holding at ∼10 GPa and 295 K, the sample color turns from silver to dark blue. It has also consumed a considerable amount of H_2_ (N_2_ 1%) gas mixture according to sample chamber shrinkage (Fig. 2a). The Raman spectra show that no signal from the initial pure lutetium remains and a few new peaks emerge most pronouncedly between 100 cm^−1^ and 200 cm^−1^ (Fig. 2b). The overall resistance also increases obviously (Fig. 2c). More interestingly, an abrupt resistance change is consistently observed at ∼250 K during warming (Fig. 2c) and at ∼200 K during cooling (Fig. 2e) with obvious hysteresis at different magnetic fields. However, no zero resistance is obtained. All the resistance-temperature curves show parallel linear trends below ∼200 K. After the reaction, the sample becomes much less conductive than the initial pure lutetium metal over the entire testing temperature range. If we follow the resistance data processing method in Ref. [16], after subtracting a linear background (a linear fit to the data below ∼200 K although it has not been proven to be scientifically justified [28]) and normalization to the resistance values at 300 K for all the raw resistance data under different magnetic fields (Fig. 2d), the transitions at ∼250 K show a seemingly gradual magnetic-field suppression behavior (Fig. 2d), which is usually a characteristic of a superconducting transition and seems to reproduce the results reported in Ref. [16]. The abrupt resistance change is also observable at ∼3.5 GPa during decompression, see Supplementary Fig. S1. In contrast to the inscrutable low success rate (35%), with our experimental protocol, the sudden resistance change has also been repeatedly reproduced in the dark blue samples without failure at different experimental conditions but only at the relatively early stage of the reaction (Supplementary Fig. S2).

It should be noted that the continuous increase of the overall resistance data (upshift of all curves in Fig. 2c) as a function of the magnetic field is very unusual for superconducting materials. This phenomenon typically suggests prominent magnetoresistance in the sample over the entire testing temperature range. However, up-shift of the overall resistance also exists during zero-field cooling from 300 K to 2 K (Fig. 2e). Following the same data processing method in Fig. 2d, the suppression-like behavior of the transition also appears (Fig. 2f) even without magnetic fields. Therefore, there is no explicit evidence to support the magnetic-field suppression effect on the resistance transition, thus lacking another critical characteristic of superconductivity. An alternative mechanism could be continuous reactions occurring in the sample during resistance measurements, resulting in an overall resistance increase and changes in the transition width (broadening) and transition temperature (left shift) with time.

**Figure 3.**
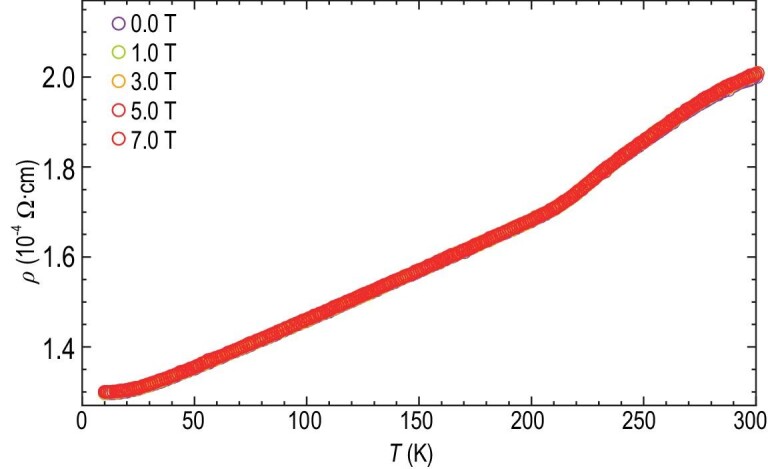
Temperature dependence of resistivity of a synthesized Lu-H-N sample recovered to ambient pressure during warming from 10 K to 300 K with different magnetic fields. The testing Lu-H-N sample was synthesized by the reaction between the lutetium foil sample and H_2_ (N_2_ 1%) gas mixture in a DAC holding at ∼2 GPa and 343 K for 4 days. No magnetic-field suppression effect on the resistivity transition exists.

Furthermore, when another reacted sample (synthesized at ∼2 GPa and 343 K for 4 days) showing a near-room temperature resistance transition is recovered to ambient pressure by fully releasing the H_2_ (N_2_ 1%) gas mixture, the resistance transition remains, which is consistent with the recent claim of ‘superconductivity’ at ambient pressure in the patent application related to Ref. 16 [34], but exhibits no shift at all with magnetic fields, as shown in Fig. 3. In this case, without the interference of a continuous chemical reaction between the sample and surrounded H_2_ (N_2_ 1%) gas mixture, the absence of magnetic-field suppression behavior explicitly excludes the possibility of superconductivity as the mechanism for the near-room temperature resistance transition.

With real-time *in situ* resistance measurements using PPMS during the reaction between the lutetium foil with H_2_ (N_2_ 1%) gas mixture explored in a broad pressure and temperature space, it is further clarified that the reaction rate (kinetics) is susceptible to both pressure and temperature. At relatively low temperatures or pressures, the reaction could be very sluggish. Eventually, the reaction leads to the formation of a final stable insulating phase with continuous resistance increases of up to 8 orders of magnitude (Supplementary Fig. S3). This conclusion is also supported by the temperature coefficient of resistance changes from positive to negative values during temperature scanning (Supplementary Fig. S4). The final insulating state shows no superconducting signal during cooling from 373 K to 2 K (Supplementary Fig. S4). It is well-known that the LuH_2_ phase is metallic and only the stoichiometric LuH_3_ phase is insulating. Thus, the final insulating state observed in Supplementary Figs S3 and S4 should be a LuH_3_-like phase. The samples shown in Figs 2 and 3, with relatively low resistance (metallic states), should be still at the early stage of their reactions, i.e. intermedium states with non-stoichiometric compositions with the H/Lu atomic ratio considerably less than 3. The continuous nature of the reaction and composition change perfectly rationalize the gradual increase of their resistance during measurements as a function of time (or the number of temperature scans) and the variation in resistance transition width, which is also a serious concern of the data in Ref. 16 [35]. In another experiment with multiple temperature scans, a gradual transition from a normal metallic state to an intermedium state with an emerged resistance upsurge at ∼230 K, and then to an insulating/semiconducting state can be observed (Supplementary Fig. S5).

Then, the question is: what causes the electrical resistance upsurge near room temperature in the Lu-H-N samples? Actually, the early lanthanides (L) have been well-known to form non-stoichiometric hydrides with quite a wide range of compositions from LH_1.9_ to LH_3_ [36]. Their dihydrides are metallic and have a cubic fluorite structure. During reaction, hydrogen will first occupy the tetrahedral interstitial sites, then, further increase of hydrogen content will result in filling the octahedral interstitial sites with slight tetragonal distortion (atomic displacement) of the lanthanide sublattice, eventually leading to the formation of insulating trihydrides [37]. A reversible metal-to-semiconductor transition with dramatic resistance upsurge has been extensively observed near room temperature (200–260 K) in substoichiometric lanthanide trihydrides [38–41]. The mechanism is associated with the localization of the defect band at Fermi energy (*E*_F_) due to temperature-dependent structural modification in substoichiometric lanthanide trihydride during warming, e.g. an order-to-disorder transition of the octahedral vacancies with superlattice of octahedral vacancies formation at low temperatures and breaking down at high temperatures [38,39]. Previous reports have confirmed that LuH_2±x_N_y_ also has a cubic fluorite structure [16,17]. Given the common crystal structure and similar resistance transitions occurring at almost the same temperature range (200–260 K), it is expected that the reacted Lu-H-N sample in this work may share the same metal-to-semiconductor/insulator transition phenomena and mechanism with the early lanthanides non-stoichiometric hydrides.

**Figure 4.**
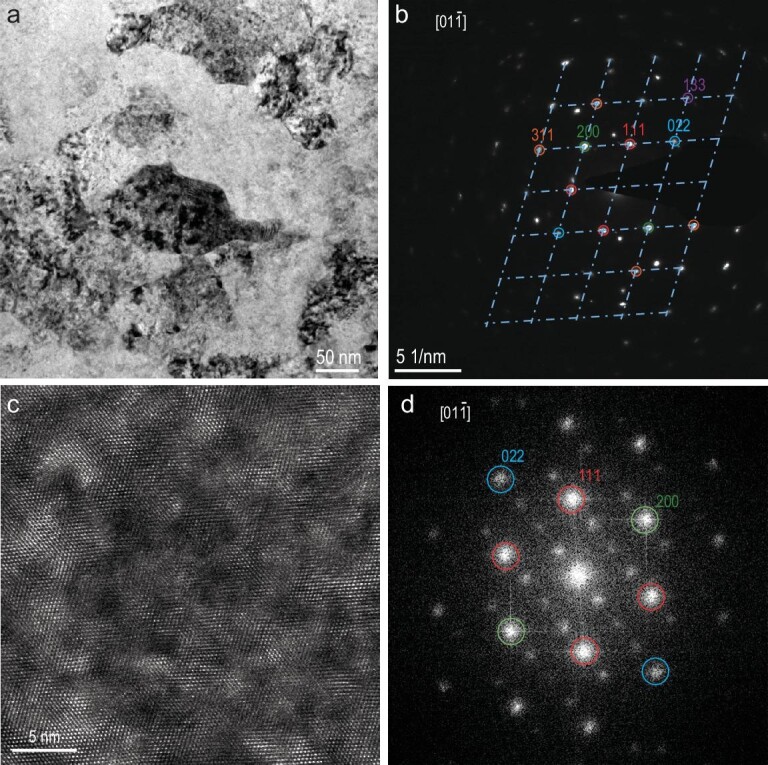
TEM characterization of the lutetium sample after reaction with the H_2_ (N_2_ 1%) gas mixture at ∼10 GPa and 343 K for 5 hours. A sudden resistance change was confirmed (as shown in Fig. S2a) before the sample was recovered to ambient conditions and sent for FIB cutting. (a) TEM image. (b) SAED image of the whole area in image (a). The SEAD pattern could be indexed into the [01$\bar{1}$] zone axis pattern of a face-centered cubic (fcc) structure with the unit cell parameter of ∼5.05 Å as denoted by the lattice of dashed lines. Extra diffraction spots in (b) are confirmed to belong to other fcc grains with identical structures. Circles with different colors highlight the different crystal planes $( {{\mathrm{hkl}}} )$. (c) HRTEM image of the center area in (a). (d) FFT image of the image in (c). Besides the spots belonging to the fcc structure, superstructure reflections are present, supporting the existence of modulated structures, which are invisible in (b) probably due to too strong diffraction signals from the fcc lattice.

By carrying out TEM measurements on a reacted lutetium sample (Fig. 4), which is confirmed to have the resistance upsurge at ∼250 K (Supplementary Fig. S2a), we reveal that the recovered phase is LuH_2±x_N_y_ (space group: Fm$\bar{3}$m, lattice parameter: ∼5.05 Å, close to the previous experimental and simulation results of the Lu-H-N samples [17,18,24,42–44]) according to the selected area electron diffraction (SAED) pattern (Fig. 4b). More importantly, in Fig. 4d, superstructure reflections besides those from the Fm$\bar{3}$m space group could be observed by Fourier transform images of the high-resolution TEM (HRTEM) image along the [01$\bar{1}$] zone axis (Fig. 4c), with modulation wave vectors of q* = 1/4 (022) and q* = 1/2 (200). Meanwhile, a ‘stripe-pattern’ could be observed from the corresponding HRTEM image. The TEM result suggests the presence of modulated structures, which can be associated to Lu/H atomic displacement or distortion of hydrogen octahedra induced by hydrogen insertion/vacancies, therefore, providing a reasonable structural basis for the metal-to-semiconductor/insulator transition scenario [45]. According to the previous results in substoichiometric lanthanide trihydrides [38–41], the superstructure reflections become unstable when approaching room temperature, which is consistent with our observation of the inhomogeneous (incomplete disappearance) feature of the superstructure reflections observed by TEM at room temperature (Supplementary Fig. S6). In addition, it should be noted that the *in situ* Raman spectra of all the reacted samples studied in this work are more consistent with the feature of the stoichiometric LuH_3_ (a few overlapped peaks below 200 cm^−1^) rather than the stoichiometric LuH_2_ phase (a characteristic peak at ∼250 cm^−1^) [17,46], which could suggest the samples synthesized in this work are more like (N-doped) substoichiometric lanthanide trihydride, LuH_3-δ_N_y_. It is suggested that the hydrogen vacancies and their order/disorder distribution as a function of temperature and pressure are critical to understanding the properties of the Lu-H-N system, which should be paid more attention to in future calculations. In addition, there is no obvious pink color observed in all samples in the pressure range explored in this work, which is in line with the simulation results for LuH_3_ [47]. Our results suggest that the sudden electrical resistance change near room temperature observed in Lu-H-N is not necessarily associated with the pink color as reported in Ref. [16], which is consistent with the previous observation of pressure-induced color change but without resistance transitions [17,20,23,26,47].

## CONCLUSION

In summary, by taking a well-controlled approach of *in situ* resistance measurements of lutetium foil during its reaction with H_2_ (N_2_ 1%) gas mixture at various pressure, temperature, and reaction time conditions, it is confirmed that the abrupt resistance change at ∼250 K could be repeatedly reproduced by careful control of the reaction time at a given temperature and pressure condition, which requires real-time monitoring of the sample resistance during the reaction. However, lacking both zero-resistance and magnetic-field suppression effect on the resistance transition rules out the possibility of relating the observed sudden resistance change to any superconducting transition. Instead, a metal-to-semiconductor/insulator transition in an intermedium state of the reaction with a non-stoichiometric composition, LuH_3-δ_N_y_, is suggested to account for the near room temperature resistance upsurge. The metal-to-semiconductor/insulator transition seems general in lanthanide hydrides, and therefore it would be more prudent to treat the low-temperature linear dependence of resistance as a meaningless system background and attribute their resistance jumps near room temperature to any superconductivity.

## METHODS

The details about the sample synthesis and characterization are included in the Supplementary data.

## Supplementary Material

nwad337_Supplemental_File
