# Supplementary material for: Origin of the near-room temperature resistance transition in lutetium with H2/N2 gas mixture under high pressure
Source: Natl Sci Rev. 2023 Dec 30;11(7):nwad337. doi: 10.1093/nsr/nwad337 (PMC11173200; doi:10.1093/nsr/nwad337)
Supplement: nwad337_Supplemental_File [file nwad337_supplemental_file.docx]

**Supplementary Data**

**for**

**Origin of the near-room temperature resistance transition in** **lutetium** **with H_2_/N_2_ gas mixture under high pressure**

Di Peng^1,2,3^, Qiaoshi Zeng^3,4,*^, Fujun Lan^3^, Zhenfang Xing^3,5^, Zhidan Zeng,^3^ Xiaoxing Ke,^6^ Yang Ding^3^, Ho-kwang Mao^3,4,*^

1. *Key Laboratory of Materials Physics, Institute of Solid State Physics,* *Hefei Institutes of Physical Science (HFIPS), Chinese Academy of Sciences, Hefei 230031, China*
2. *Science Island Branch, Graduate School of University of Science and Technology of China, Hefei 230026, China*
3. *Center for High Pressure Science and Technology Advanced Research, Shanghai 201203, China*
4. *Shanghai Key Laboratory of Material Frontiers Research in Extreme Environments (MFree), Shanghai Advanced Research in Physical Sciences (SHARPS), Shanghai 201203, China*
5. *State Key Laboratory of Superhard Materials, Institute of Physics, Jilin University, Changchun 130012, China*
6. *Faculty of Materials and Manufacturing, Beijing University of Technology, Beijing 100124, China.*

** Corresponding authors. E-mails:* [*zengqs@hpstar.ac.cn*](mailto:zengqs@hpstar.ac.cn)*, or maohk@hpstar.ac.cn*

**This file includes Methods and Figures. S1-S6.**

**METHODS**

**Sample synthesis and loading.** The Lu-H-N sample was prepared by a direct reaction between pure lutetium foil and H_2_ (N_2_ 1%) gas mixture at a certain pressure and temperature condition. High-purity lutetium (99.9%, Alfa Aesar) and H_2_ (N_2_ 1%) gas mixture (volume ratio is 99 to 1) were used as the raw materials for the synthesis. The lutetium thin foil with a thickness of ~10 μm was obtained by cutting and forging a block of metal lutetium. Then, square samples with a side length of approximately 80 microns were cut from the thin foil. High-pressure chemical reactions and resistance measurements were conducted in a customized Be-Cu alloy DAC with a culet size of ~300 µm using a non-magnetic metal (rhenium) gasket. The rhenium gasket was pre-indented to ~20 GPa. The bottom of the indent of the rhenium gasket was then removed by laser drilling and refilled with a mixture of cubic boron nitride and epoxy resin as the insulating material for electrical measurements. The sample chamber is a hole with a diameter of ~300 µm and a thickness of ~30 µm drilled by laser in the pre-indented filled mixture of cubic boron nitride and epoxy resin. Ruby balls are loaded with the sample as a pressure calibrant.

**In situ resistance measurement.** Four platinum electrodes were arranged symmetrically on the Lu sample to monitor the resistance variation during its chemical reaction with H_2_ (N_2_ 1%) gas mixture. The gas mixture was loaded into the high-pressure Be-Cu DAC using a gas loading system. The Be-Cu DAC with four platinum electrodes is connected to the PPMS instrument for *in situ* resistance measurements on the sample during reaction at different pressure and temperature conditions.

**Raman spectroscopy.** Since our samples for *in situ* resistance measurements are loaded in a Be-Cu DAC with a too-small angular opening for x-ray diffraction measurement, we mainly employed Raman spectroscopy instead to determine the phase evolution during the reaction between lutetium foil with H_2_ (N_2_ 1%) gas mixture at various pressure and temperature conditions. Raman scattering measurements were carried out on an in-house system equipped with a Charge Coupled Device and Spectrometer (Princeton Instruments), which used a laser with a wavelength of 488 nm and a power of 5 mW at room temperature.

**TEM measurements.** For the TEM measurements, the reacted sample with a sudden resistance change near room temperature was synthesized by reaction at ~10 GPa and 343 K for 5 hours. Then, samples were cut using a focused ion beam (FEI Versa 3D). Transmission electron microscopy (TEM) images and selected area electron diffraction (SAED) patterns were obtained using a Tecnai F20 (FEI) TEM at an accelerating voltage of 200 kV.


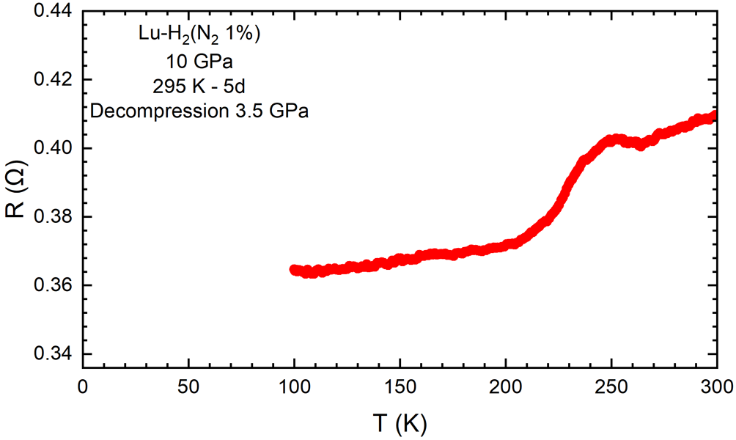


**Figure S1** Temperature dependence of resistance for the lutetium sample immersed in the H_2_ (N_2_ 1%) gas mixture at ~3.5 GPa during decompression from ~10 GPa after the completion of all the measurements shown in Fig. 2. The data were collected during the warning process. The sudden resistance change at ~250 K remains.





**Figure S2** Temperature dependence of resistance for lutetium sample immersed in the H_2_ (N_2_ 1%) gas mixture after reactions at different experimental conditions: (a) ~10 GPa and 343 K for 5 hours; (b) ~10 GPa and 373 K for 0.5 hours; (c) ~5 GPa and 353 K for 2.5 hours. Although a sudden resistance change is present in all three figures, the differences in magnitude, position, and width of the resistance change indicate the sensitivity of the reaction to experimental conditions.

**
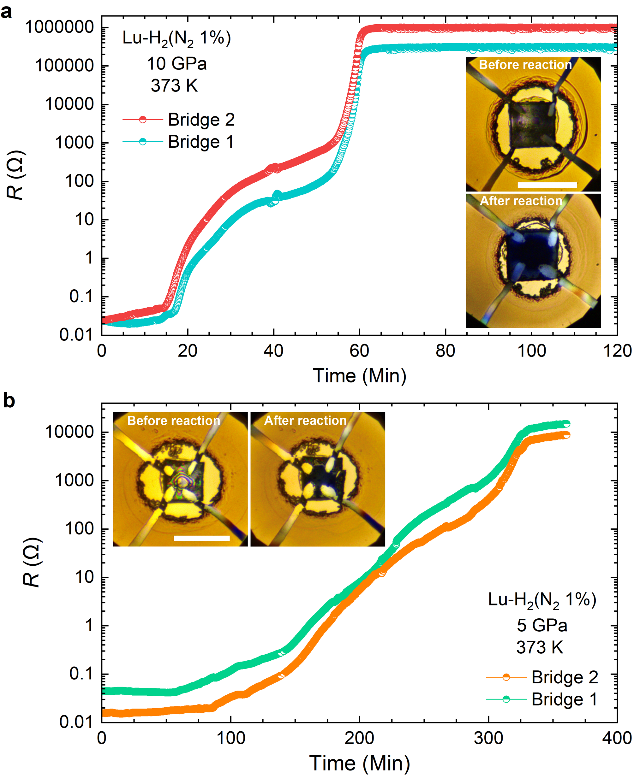
**

**Figure S3** Time dependence of electrical resistance for the lutetium foil sample loaded in H_2_ (N_2_ 1%) gas mixture in a DAC at 10 GPa, 5 GPa and 373 K. (a) Reaction time dependence of resistance of the lutetium foil sample immersed in H_2_ (N_2_ 1%) gas mixture at ~10 GPa and 373 K. The insets show the images of lutetium foil samples with four platinum electrodes in H_2_ (N_2_ 1%) gas mixture before (upper) and after (bottom) the reaction. The sample resistance quickly reaches a plateau after only 60 minutes, with the sample color changed from silver eventually to dark blue (inset of Fig. 3a), suggesting the completion of the reaction with the resistance increase by ~8 orders of magnitude. (b) Reaction time dependence of resistance of the lutetium foil sample immersed in H_2_ (N_2_ 1%) gas mixture at ~5 GPa and 373 K. The metal-to-insulator transition completes at ~325 mins with a resistance increase of ~6 orders of magnitude. The insets show the images of lutetium foil samples with four platinum electrodes in H_2_ (N_2_ 1%) gas mixture before (left) and after (right) reaction. With identical reaction temperatures, it is clear that lower pressure slows down the reaction and decreases the saturated resistance of the insulator state. The scale bars in the insets present 100 μm.


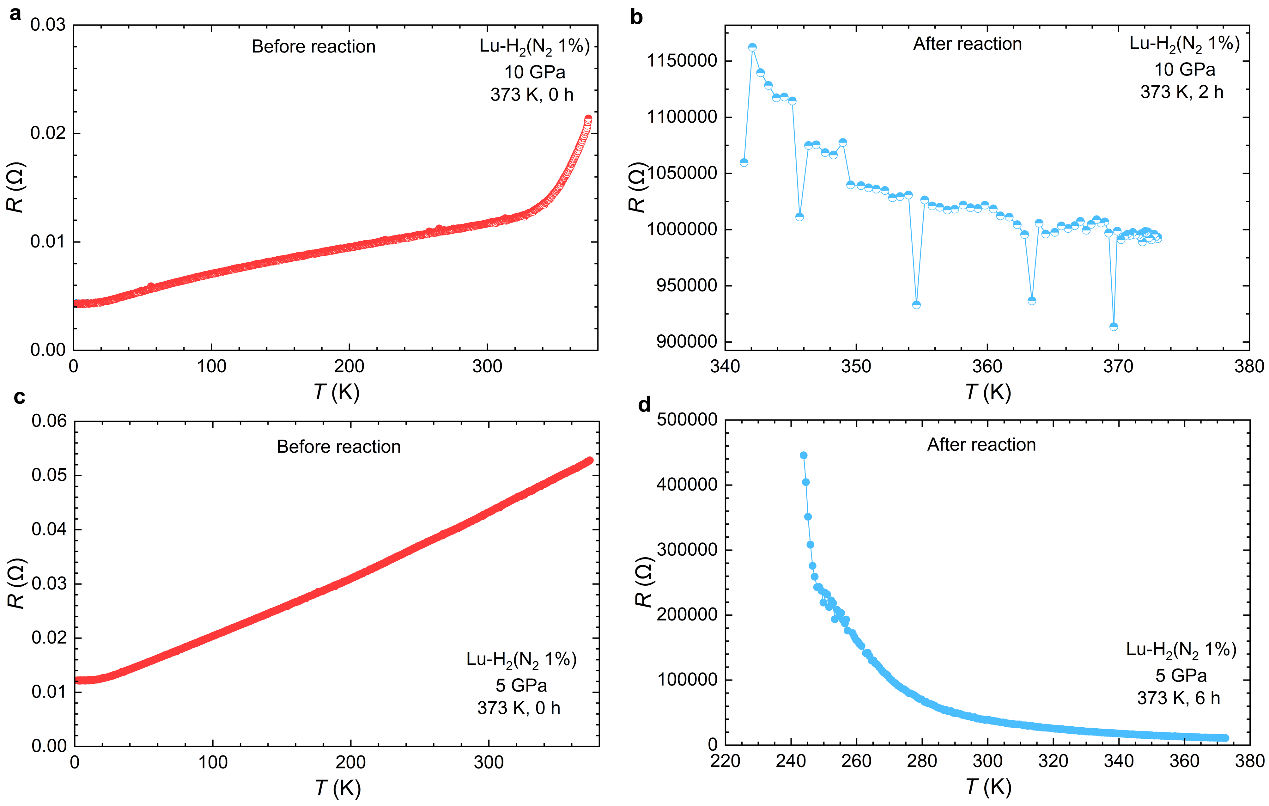


**Figure S4** Temperature dependence of resistance for lutetium sample immersed in the H_2_ (N_2_ 1%) gas mixture before and after reaction at different experimental conditions. (a) Before reaction at ~10 GPa during warming from 2 K to 373 K. The sharp slope increase above ~340 K indicates that an obvious reaction takes place above this temperature at ~10 GPa. (b) After reaction at ~10 GPa and 373 K for 2 hours. A typical insulator behavior with the negative temperature dependence of resistance presents. (c) Before reaction at ~5 GPa during warming from 2 K to 373 K. (d) After reaction at ~5 GPa and 373 K for 6 hours. A typical insulator behavior with the negative temperature dependence of resistance presents.

**

**

**Figure S5** Temperature dependence of resistance for lutetium foil sample immersed in the H_2_ (N_2_ 1%) gas mixture at ~5 GPa during warming between 2 K and 353 K before (2 GPa, 295 K, 0.0 h, a) and after the reaction at 353 K (80 ℃) for different elapsed time (multiple temperature scans, b-f). Each temperature scan from 353 K to 10 K takes ~0.5 hour. A sudden resistance change could be reproduced at the early stage of the reaction with a gradual increase of the overall resistance even when the entire sample shows a semiconductor or insulator behavior with a negative temperature dependence of resistance (f). The resistance upsurge at ~230 K remains even in the early stage of the insulator/semiconducting state, which indicates the resistance upsurge could exist in a relatively wide compositional range.


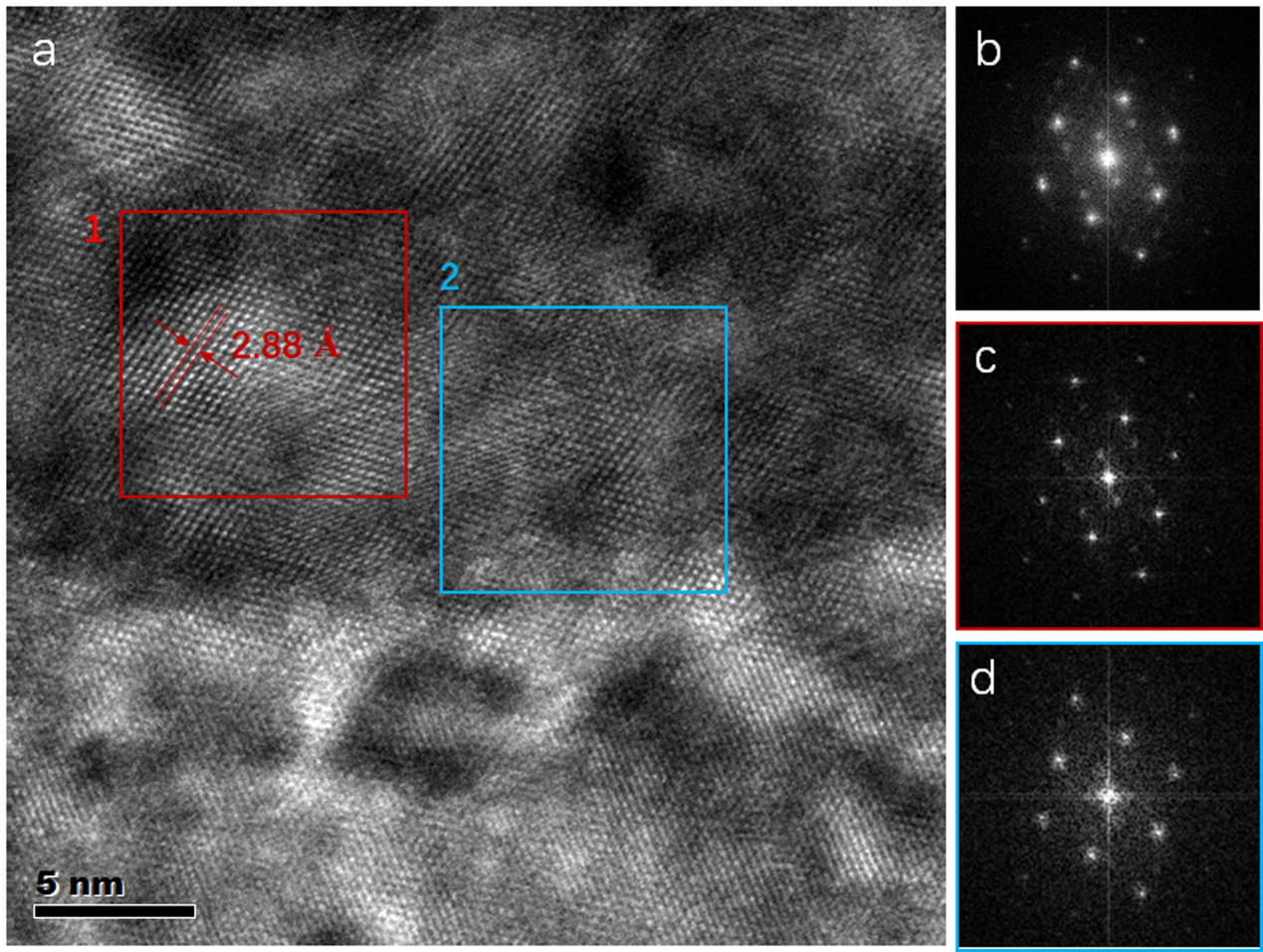


**Figure S6** TEM characterization of the lutetium sample after reaction with the H_2_ (N_2_ 1%) gas mixture at ~10 GPa and 343 K for 5 hours. A sudden resistance change was confirmed (as shown in Fig. S2a) before the sample was recovered to ambient condition and sent for FIB cutting. (a) A typical HRTEM image. (b) FFT image of the whole area in image (a), which can be indexed into the [$01\overline{1}$] zone axis pattern of an fcc structure. Besides the spots belonging to the fcc structure, relatively weak superstructure reflections are present, suggesting the existence of modulated structures. However, the existence of modulated structure is not homogeneous. The FFT images (c) and (d) are from the areas selected by the red square 1 and the blue square 2 in (a), respectively. The pattern in (c) shows superstructure reflections, but the pattern in (d) only has reflections belonging to the fcc structure.
